# Inhibition of PIKfyve by YM-201636 Dysregulates Autophagy and Leads to Apoptosis-Independent Neuronal Cell Death

**DOI:** 10.1371/journal.pone.0060152

**Published:** 2013-03-27

**Authors:** Sally Martin, Callista B. Harper, Linda M. May, Elizabeth J. Coulson, Frederic A. Meunier, Shona L. Osborne

**Affiliations:** The University of Queensland, Queensland Brain Institute, Brisbane, Queensland, Australia; University of Iowa, United States of America

## Abstract

The lipid phosphatidylinositol 3,5-bisphosphate (PtdIns(3,5)*P*_2_), synthesised by PIKfyve, regulates a number of intracellular membrane trafficking pathways. Genetic alteration of the PIKfyve complex, leading to even a mild reduction in PtdIns(3,5)*P*_2_, results in marked neurodegeneration via an uncharacterised mechanism. In the present study we have shown that selectively inhibiting PIKfyve activity, using YM-201636, significantly reduces the survival of primary mouse hippocampal neurons in culture. YM-201636 treatment promoted vacuolation of endolysosomal membranes followed by apoptosis-independent cell death. Many vacuoles contained intravacuolar membranes and inclusions reminiscent of autolysosomes. Accordingly, YM-201636 treatment increased the level of the autophagosomal marker protein LC3-II, an effect that was potentiated by inhibition of lysosomal proteases, suggesting that alterations in autophagy could be a contributing factor to neuronal cell death.

## Introduction

Phosphoinositides are important lipid regulators of membrane trafficking and cellular signalling. Phosphatidylinositol 3,5-bisphosphate (PtdIns(3,5)*P*_2_) is synthesised by the Class III PtdIns-5-kinase, PIKfyve. PIKfyve is part of an active complex regulating PtdIns(3,5)*P*_2_ levels, which includes the lipid phosphatase Fig4 and accessory protein Vac14 [1], [2]. Despite being a minor component of cellular lipids, PtdIns(3,5)*P*_2_ and/or PIKfyve has been implicated in many cellular processes, including trafficking through the endolysosomal system, exocytosis, ion channel regulation and autophagy [3]–[10]. In mice, genetic ablation of PIKfyve results in pre-implantation lethality [11], while mutations or genetic ablation of Fig4 or Vac14 results in decreased levels of PtdIns(3,5)*P*_2_ and a prominent vacuolar phenotype in the central nervous system, accompanied by marked spongiform degeneration [12], [13]. In humans, mutations in Fig4 are causative of Charcot-Marie-Tooth disease type 4J [12] and are associated with forms of amyotrophic lateral sclerosis [14]. Targeted re-expression of Fig4 in neurons of Fig4^−/−^ mice clearly demonstrates a primary role for neuronal PIKfyve activity in preventing spongiform degeneration [15].

Despite the above lines of evidence, little is known of the mechanisms underlying neuronal cell death in response to disruption of PIKfyve activity. In Fig4 and Vac14 mutant mice, neuronal cell death appears to be preceded by cellular vacuolation [12], [13]. Moreover, cultured Fig4^−/−^ cerebellar neurons are highly vacuolated. This vacuolation is reminiscent of that observed in non-neuronal cell lines, where interfering with PIKfyve activity results in the swelling of endocytic compartments and disruption of endomembrane transport [3], [6], [16], [17]. Macroautophagy (hereafter referred to as autophagy) has been implicated in neuronal survival and in the pathogenesis of a number of neurodegenerative diseases [18], [19]. Furthermore, in the Fig4^−/−^ mouse brain, an increase in the levels of the autophagy marker protein LC3-II, together with the autophagy chaperone protein p62, has been interpreted as a block in the completion of autophagy [20]. Interestingly, targeted re-expression of Fig4 in glia, but not neurons, of Fig4^−/−^ mice, prevents the accumulation of autophagy markers but does not rescue the spongiform degeneration [15]. To date, the importance of PIKfyve activity in facilitating autophagy in neurons remains unclear.

To provide further insight into the mechanism by which reduced levels of PtdIns(3,5)*P_2_* leads to neuronal cell death, we used the PIKfyve inhibitor YM-201636 [6], at a concentration known to induce cellular vacuolation through an effect on PtdIns(3,5)*P_2_* levels [21]. We demonstrate that directly inhibiting PIKfyve kinase activity causes vacuolation and neuronal cell death via a caspase-independent mechanism, and is associated with alterations in autophagy. Our data point to a fundamental requirement for PtdIns(3,5)*P*_2_ in the survival and development of hippocampal neurons, and suggest that alterations in the regulation of the autophagosomal system could contribute to the mechanism of neuronal cell death observed upon PIKfyve inhibition.

## Materials and Methods

### Antibodies and Reagents

Antibodies were obtained from the following sources: rabbit anti-LC3 (Novus Biologicals, NB100-2331), mouse anti-ß-actin (Sigma, S0644), mouse anti-ßIII tubulin (Covance, MMS-435P), rabbit anti-EEA1 (#2411) and rabbit anti-cleaved caspase-3 (#9661) (Cell Signaling Technology), mouse anti-LAMP1/LY1C6 (Sapphire Bioscience, #120-13523), rat anti-LAMP1/CD107a (#553792), and mouse anti-GM130 (#610822) (BD Biosciences). Fluorescently (Alexa Fluor)-tagged endocytic marker proteins and secondary antibodies were obtained from Invitrogen. Remaining reagents were obtained from Sigma unless stated otherwise. ptfLC3 was generated in the laboratory of Prof Tamotsu Yoshimori, Osaka University, Japan and obtained from Addgene (Plasmid 21074; [22]).

### Cell Culture and Neuronal Survival Assays

All animals received care in compliance with the Australian code of practice for care and use of animals for scientific purposes, and all experiments carried out were done so with approval from the University of Queensland Animal Ethics Committee.

Cultured hippocampal neurons were prepared from embryonic day 18 C57BL/6 mice and cultured in 45% DMEM/45% Hams F12 containing 10% Neurocult (StemCell Technologies) and 3.75 ng/ml brain-derived neurotrophic factor (R&D Systems) [23]. Briefly, embryos were collected in ice-cold Leibowitz’s 15 medium (Gibco), the brains removed and the hippocampus from each hemisphere dissected out. Hippocampal tissue was digested with 0.05% trypsin-EDTA for 10–15 min at 37°C then neutralised with trypsin inhibitor and centrifuged at 104 g for 7 min. The cell pellet was resuspended in medium, serially triturated through 19 gauge and 23 gauge needles, and passed through a 40 µm cell strainer (BD Biosciences). Neurons were counted for viable cell number, judged on their ability to exclude trypan blue.

For neuronal survival assays each well of a 4-well 3 cm Cell-Star® plastic dish (Greiner) was etched with an 18 gauge needle to define a 10×10 grid, and the wells were coated with 0.1 mg/ml poly-L-lysine. Neurons were plated at a density of 40,000 cells per well and cultured as above except that the medium was supplemented with 2 ng/ml brain-derived neurotrophic factor. After 24 h the medium was replaced and one hour later neurons were counted using relief contrast on an inverted Olympus 618X1 light microscope with CO_2_ incubation at room temperature. The grid locations were noted for all neurons counted. Treatments were then initiated as described in the results sections and after 24 h surviving neurons were counted from the same grid locations. Surviving neurons were defined as showing no sign of blebbing or extensive vacuolation, shrunken or apoptotic soma, or defective formation of neuritic processes. Cell survival was calculated as a percentage of starting cell numbers.

PC12 cells were maintained as described previously [8]. A reporter PC12 cell line stably expressing the tfLC3 was generated by lipid-based transfection (Lipofectamine LTX) and G418 selection, and fluorescence-activated cell sorting used to enrich for cells with a low level of expression. The reporter cell line was maintained in normal PC12 growth medium supplemented with 0.5 mg/ml G418.

### Sample Preparation and Western Blotting

Primary hippocampal neurons were either solubilised directly in SDS-PAGE sample buffer containing 25 mM DTT and protease inhibitors, or solubilised in 20 mM Hepes pH 7.3, 150 mM NaCl, 2% Triton X-100 and protease inhibitors for 30 min at 4°C, insoluble material removed by centrifugation and protein concentration determined using a Bradford assay (Bio-Rad). 50 µg protein was re-suspended in SDS-PAGE sample buffer. SDS-PAGE and Western blot analysis was carried out as described previously [8]. Blots were visualised and bands were quantified using the Odyssey system (Licor).

### Immunofluorescence Microscopy

Cells were fixed in 4% paraformaldehyde in phosphate-buffered saline and processed for immunocytochemistry as described previously [24], [25]. Permeabilisation was performed using 0.1% saponin (for EEA1) or 0.05% Triton X-100 (other antibodies). Cells were imaged using a Zeiss LSM510 confocal microscope. The uptake and trafficking of endocytic probes was carried out using Alexa Fluor 555-conjugated proteins at the following concentrations: 25 µg/ml transferrin, 5 µg/ml WGA, or 1 µg/ml CTB. Cells were treated by adding 1 µM of YM-201636 or DMSO for 4 h, and supplemented with the indicated probes for either the entire 4 h duration, or the final 5 min, 30 min or 2 h of treatment, as described in the results section. Cells were then processed for immunocytochemistry as described above. Analysis was carried out in either Fiji (NIH; [26]) or Imaris (Bitplane). All images were processed using Adobe Photoshop CS3 and figures compiled with Adobe Illustrator CS3.

### Electron Microscopy

Primary hippocampal neurons were incubated in growth medium with DMSO or 1 µM YM-201636 for 4 h or 22 h respectively, rinsed briefly in PBS and fixed in 2.5% glutaraldehyde (Electron Microscopy Sciences). Fixed cells were contrasted with 1% osmium tetroxide and 4% uranyl acetate prior to dehydration and embedding in LX-112 resin [27]. Sections (50 nm) were cut using an ultramicrotome (UC64; Leica). To analyse endocytosis WGA-HRP was included in the growth medium at 10 µg/ml for the entire period of PIKfyve inhibition (4 h) or for the final 30 min, as described in the results section. Following fixation, cells were processed for 3,3′-diaminobenzidine (DAB) cytochemistry using standard protocols, prior to contrasting, dehydration and embedding as described above. All images were processed using Adobe Photoshop CS3 and figures compiled with Adobe Illustrator CS3.

To quantify vacuole formation, cell bodies with a clearly visible nucleus were visualised at 6000× using a transmission electron microscope (model 1011; JEOL) equipped with a Morada cooled CCD camera. A 1 µm square lattice grid was overlaid on the section using the iTEM AnalySIS software. Grid intersections that fell on vacuoles or cytosol were recorded and used to determine the relative vacuole area.

### Statistical Analyses

Statistical significance was determined using a 2-tailed, Student’s *t*-test assuming unequal variance, unless stated otherwise. Results are shown as mean ± SEM unless stated otherwise.

## Results

### PIKfyve Inhibition Results in Neuronal Cell Death in Primary Hippocampal Neurons

**Figure 1:**
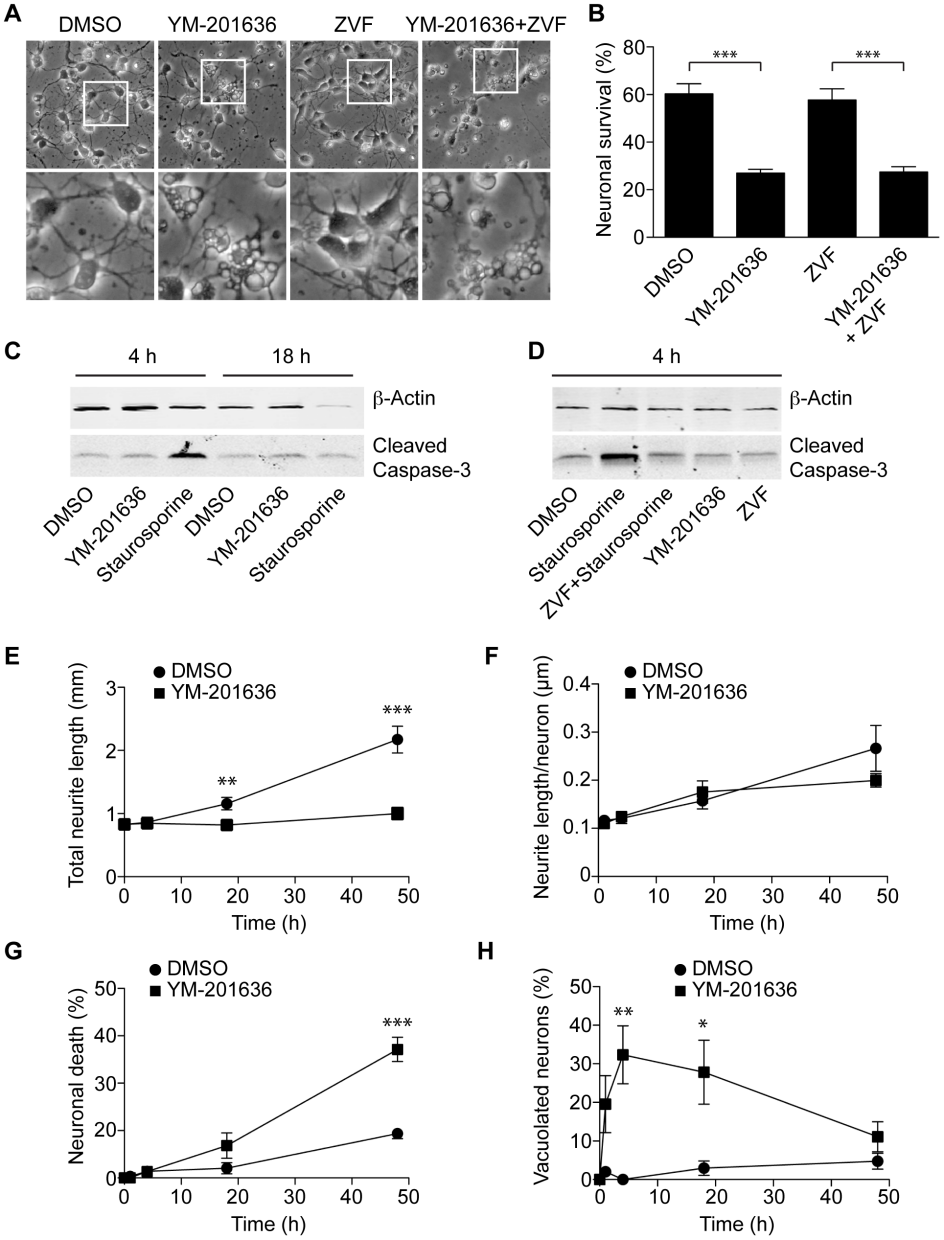
YM-201636 promotes an apoptosis-independent cell death in cultured primary hippocampal neurons. (A) Primary hippocampal neurons were treated for 24 h with DMSO or 1 µM YM-201636 with and without 30 µM Z-VAD-fmk and imaged by brightfield microscopy. (B) Quantitation of neuronal survival following 24 h treatment with DMSO or 1 µM YM-201636 with and without 30 µM Z-VAD-fmk (ZVF), n = 12 fields of cells, 3 independent experiments. (C) Primary hippocampal neurons were treated for 4 h or 18 h with DMSO, 1 µM YM-201636 or 500 nM staurosporine and immunoblotted for cleaved caspase-3 and β-actin. (D) Primary hippocampal neurons were treated for 4 h with DMSO, 500 nM staurosporine with and without 30 µM ZVF, 30 µM ZVF or 1 µM YM-201636 and immunoblotted for cleaved caspase-3 and β-actin. (E-H) Primary hippocampal neurons were imaged for 48 h in real time using brightfield microscopy and analysed for total neurite length (E) and neurite length per neuron (F) (n = 20 fields in total from 4 wells of cells) or the percentage of dead (G) or vacuolated (H) cells (n = 4 wells). All results show mean ± SEM. Circle = DMSO, Square = YM-201636. The level of significance is shown relative to DMSO, *p<0.05, **p<0.01, ***p<0.001.

Previous studies have shown that genetic manipulations of the PIKfyve complex leading to reduced PtdIns(3,5)*P*_2_ levels promotes vacuolation of neurons *in vivo* and *in vitro*, followed by neurodegeneration [1], [12], [13]. To investigate the mechanism contributing to this neuronal cell death pathway, we used the specific PIKfyve inhibitor YM-201636 in isolated hippocampal neurons. Neurons were cultured for 2 days in the presence of brain-derived neurotrophic factor prior to the addition of either DMSO (Movie S1) or 1 µM YM-201636 (Movie S2), and imaged in real time for a further 2 days. Time-lapse analysis showed that YM-201636 treatment led to vacuolation in primary hippocampal neurons and failure to develop neurite networks. Importantly, YM-201636 also promoted a decrease in the rate of neuronal survival. Quantitative measurement of cell survival after 24 h showed a marked reduction of ∼50% following PIKfyve inhibition (Figure 1A,B). Together these data suggest that acute inhibition of PIKfyve closely mimics the neuronal cell death phenotype observed in Fig4^−/−^ and Vac14^−/−^ mice [1], [12], [13]. PIKfyve inhibition also resulted in a similar phenotype (an early onset vacuolation preceding cell death) in the small population of glial cells (mostly astrocytes) present within the hippocampal cultures (Figure S1).

Apoptosis is a prevailing programmed cell death mechanism in neurons and is activated in neurodegenerative conditions such as Alzheimer’s disease [28], [29]. To determine whether neuronal cell death triggered by PIKfyve inhibition is underpinned by an apoptotic process we examined both the production of activated caspase-3 in response to YM-201636 treatment [30] and the ability of the pan-caspase inhibitor, Z-VAD-fmk, to rescue YM-201636-induced neuronal cell death (Figure 1A–D). Neuronal cultures treated for 4 h or 18 h with YM-201636 showed no increase in caspase-3 cleavage (Figure 1C). In contrast, the kinase inhibitor staurosporine, a well-established inducer of apoptosis [31], triggered a robust increase in caspase-3 cleavage within 4 h and cell death within 18 h. Caspase-3 cleavage in response to staurosporine could be blocked using Z-VAD-fmk (Figure 1D). However, Z-VAD-fmk was unable to rescue neuronal death caused by PIKfyve inhibition in the survival assay (Figure 1A–B). Z-VAD-fmk alone had no effect on neuronal survival, cell morphology or caspase-3 cleavage.

As we had also observed reduced neurites in YM-201636-treated cells, we used neurite tracing to examine the development of the neurite network (Figure 1E–F). While there was no significant difference between control and treated cells for up to 4 h, control cells subsequently developed extensive neurite networks whereas YM-201636-treated cells did not. Examination of neurite length per cell showed no significant difference between control and treated cells, indicating that individual neurons resistant to the inhibitor are able to form neurites normally. We subsequently determined the time to onset of vacuolation and cell death (Figure 1G,H). Vacuolation of the neurons first occurred within 4 h of YM-201636 addition, preceding both cell death and neurite loss, which predominantly occurred from 18–48 h. These data demonstrate that PIKfyve inhibition promotes cell death in primary hippocampal neurons through an apoptosis-independent mechanism. Furthermore, as vacuolation of neurons was detected well before neurite loss and cell death, our data suggest that altered endolysosomal function could underlie the survival defect elicited by PIKfyve inhibition.

### PIKfyve Inhibition does not Affect Endocytosis but Alters Endocytic Morphology and Endocytic Trafficking in Primary Neurons

**Figure 2:**
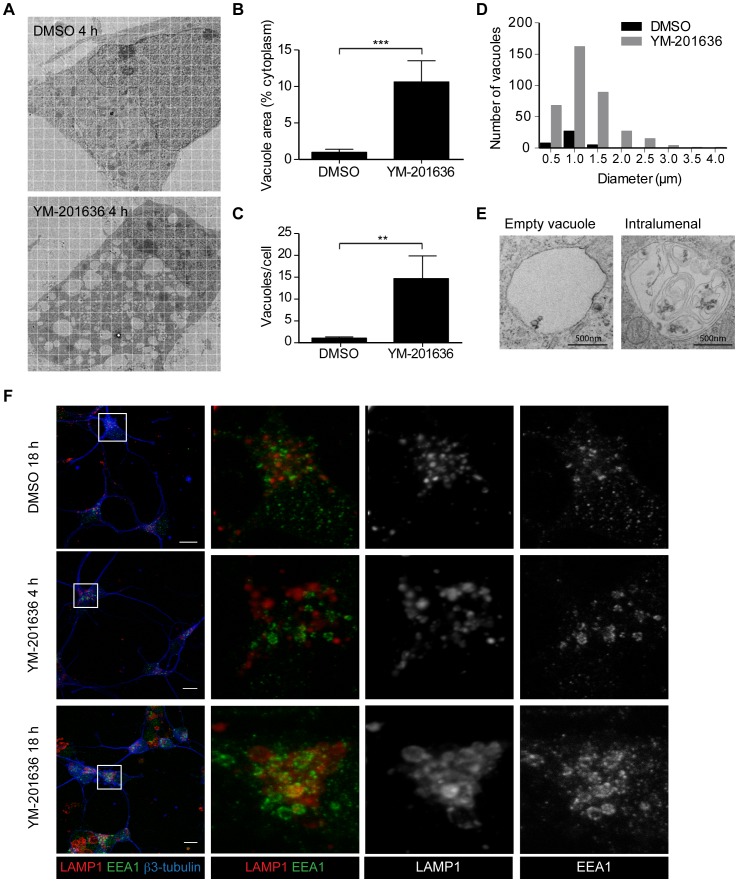
YM-201636 promotes vacuolation and endosomal compartments in hippocampal neurons. (A) Primary hippocampal neurons were treated with DMSO or 1 µM YM-201636 for 4 h and processed for electron microscopy. Images show neuronal cell bodies at the level of the nucleus, demonstrating vacuolation in the presence of YM-201636. Grid size = 1 µm square. (B-D) Electron microscopic analysis of the vacuole area as a percentage of total cytoplasmic area (B) and the number of vacuoles per cell (C) shows a significant increase in vacuole size and number following 4 h treatment with 1 µM YM-201636, **p<0.01, ***p<0.001. (D) Histogram of number of vacuoles relative to their size (representative experiment), n = 12 (DMSO) or 11 (YM-201636) cells. (E) Examples of vacuole phenotypes detected in primary hippocampal neurons treated with 1 µM YM-201636 for 4 h. (F) Primary hippocampal neurons were treated with DMSO or 1 µM YM-201636 for 4 h or 18 h and immunolabelled for LAMP1, EEA1 and ß3-tubulin. 3D projections are shown. Scale bar = 10 µm.

Previous studies have shown that deficiencies in PIKfyve activity can affect different aspects of endosomal trafficking, including transport to and from the late endosomal/lysosomal system, autophagy and endocytosis [3], [6], [32]. Analysis of the time-lapse imaging of neurons had demonstrated that the earliest detectable phenotype, preceding cell death, was the appearance of enlarged vacuoles. The nature of these vacuoles was initially examined by electron microscopy. Analysis of control and YM-201636-treated neurons demonstrated that PIKfyve inhibition for 4 h increased both the number and size of electronlucent vacuoles in the cell body and in neurites (Figure 2A–D). Quantification of vacuole formation revealed that the cell body of control neurons contained from 0–6 vacuoles, ranging from 200–1500 nm in diameter, with the average total vacuole area comprising <1% of the cell body. In contrast, the cell bodies of YM-201636-treated neurons contained an average of 14–18 vacuoles/cell, ranging in diameter from 200–>4000 nm, and occupying an average of 10–13% of the cell body (Figure 2B–D), although more highly vacuolated cells were also observed (Figure 2A). Closer examination of vacuoles formed following PIKfyve inhibition revealed varying morphological features. Although the majority of vacuoles appeared largely devoid of internal structures or only contained a few small internal vesicles [16], approximately one third (35.85±3.09%, n = 4 independent experiments, 6–12 cell profiles quantified/experiment) contained intralumenal membranes and electron-dense material, including membrane-bound intralumenal cytosolic inclusions, consistent with an autophagic component (Figure 2E [33]). To examine the possible effect of PIKfyve inhibition on the formation of autophagosomes, the number of morphologically identifiable canonical autophagic compartments was quantified. The morphological criteria used to classify the compartments are detailed in Figure S2. YM-201636 had no effect on the average number of immature or degradative autophagosomes, suggesting that inhibition of PIKfyve does not prevent autophagosome formation. However, there was a highly significant decrease in the number of electron-dense lysosomes detected (3.33±0.38/cell profile in DMSO-treated cells vs. 0.60±0.21/cell profile in YM-201636-treated cells, n = 4 independent experiments, 6–12 cell profiles/experiment, p<0.001). Together with the observed phenotypes of the YM-201636-induced vacuoles, these data suggest that inhibiting PIKfyve predominantly effects the maturation of lysosomes and autolysosomes. This is in good agreement with data from studies of PIKfyve function in *C. elegans* [32], pointing to the accumulation of enlarged pre-lysosomal autophagic and endocytic organelles.

To confirm that the vacuoles detected in neurons were derived from the endolysosomal system, we analysed the localisation of the early endosomal protein EEA1 and the lysosomal marker LAMP-1 (Figure 2F) by immunofluorescence microscopy. While PIKfyve inhibition resulted in a clear increase in the size of both compartments, the majority of vacuoles were labelled for LAMP-1, consistent with a dominant effect on lysosomes. Since PIKfyve is known to aid in regulating traffic in the endosomal system, we wanted to know whether there was a loss of identity between early and late endosomes. The correlation between EEA1 and LAMP1 labelling was therefore examined using Pearson’s correlation coefficient. Consistent with the localisation of these two proteins to distinct intracellular compartments, in control cells we found a very low level of correlation (0.06±0.01). Following treatment with YM-201636 we found that this correlation was slightly decreased (-0.02±0.03 and −0.04±0.02 in 4 h and 18 h YM-201636-treated cells, respectively, n = 3 independent experiments, 6 images/experiment). This data suggest that following inhibition of PIKfyve the identity of early and late endosomes is maintained, and the reduction in correlation detected can be most likely attributed to the redistribution of late endosomes and lysosomes from the perinuclear region to a more dispersed peripheral localisation [34].

**Figure 3:**
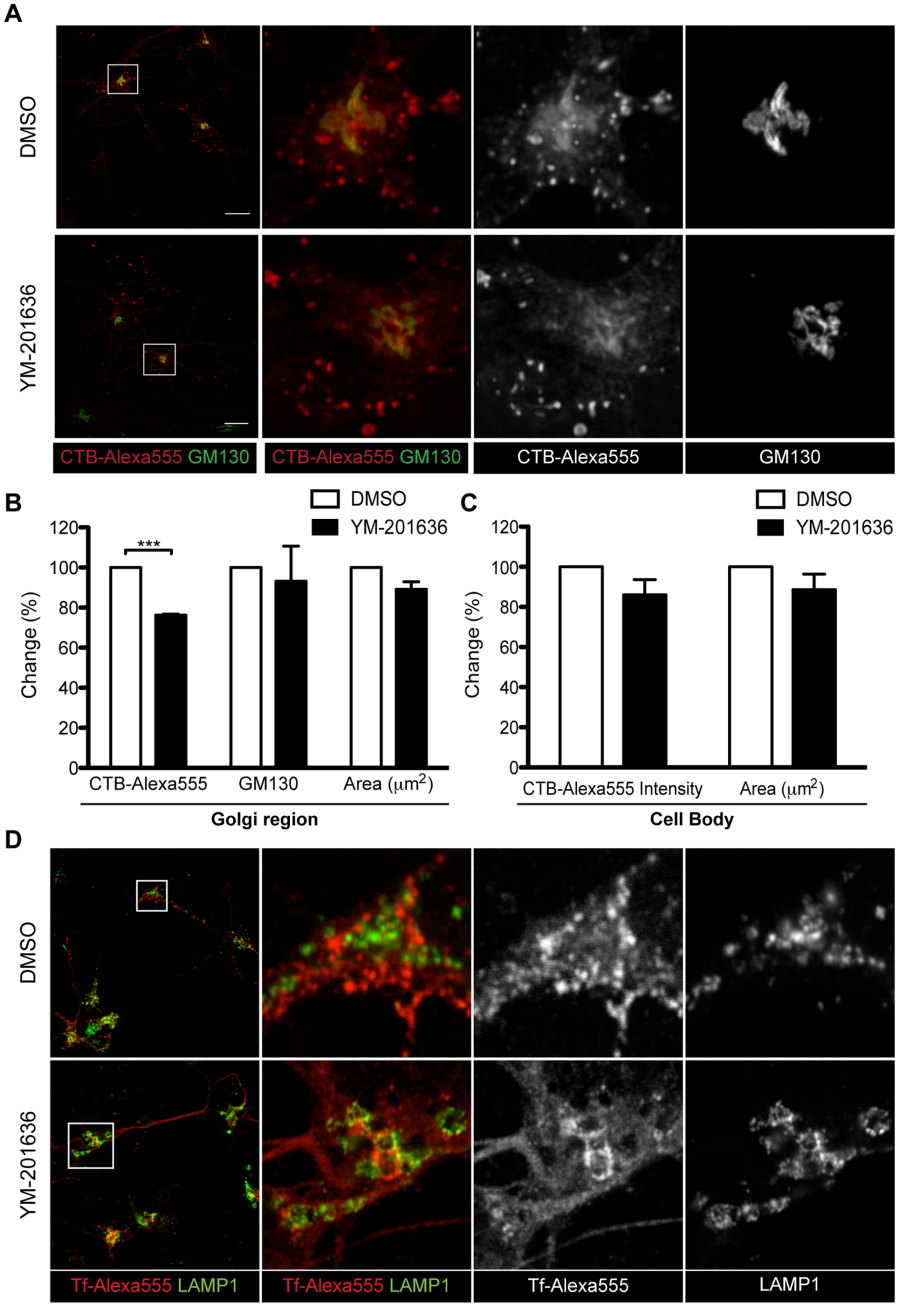
The effect of YM-201636 on endosomal and retrograde trafficking in hippocampal neurons. (A) Primary hippocampal neurons were treated with DMSO or 1 µM YM-201636 for 2 h then supplemented with 1 µg/ml CTB-Alexa555 for a further 2 h. Cells were fixed and immunolabelled for GM130. Representative 3D projections are shown. (B) The integrated intensity (per µm^2^) of CTB-Alexa555 in Golgi complex, as defined by GM130, the integrated intensity of GM130 and the size of the area analysed was measured and the percentage change between conditions determined. (C) The change in total CTB-Alexa555 integrated intensity within the cell body and the area (µm^2^) of the cell body was determined. (mean ± SEM, n = 3 independent experiments, 11–20 cells per experiment). Significances relative to DMSO *p<0.05, **p<0.01, ***p<0.001 (D) Primary hippocampal neurons were treated with DMSO or 1 µM YM-201636 for 3.5 h then supplemented with 25 µg/ml transferrin-Alexa555 (Tf-Alexa555) for a further 30 min. Cells were fixed and immunolabelled for LAMP1. Scale bar = 10 µm.

**Figure 4:**
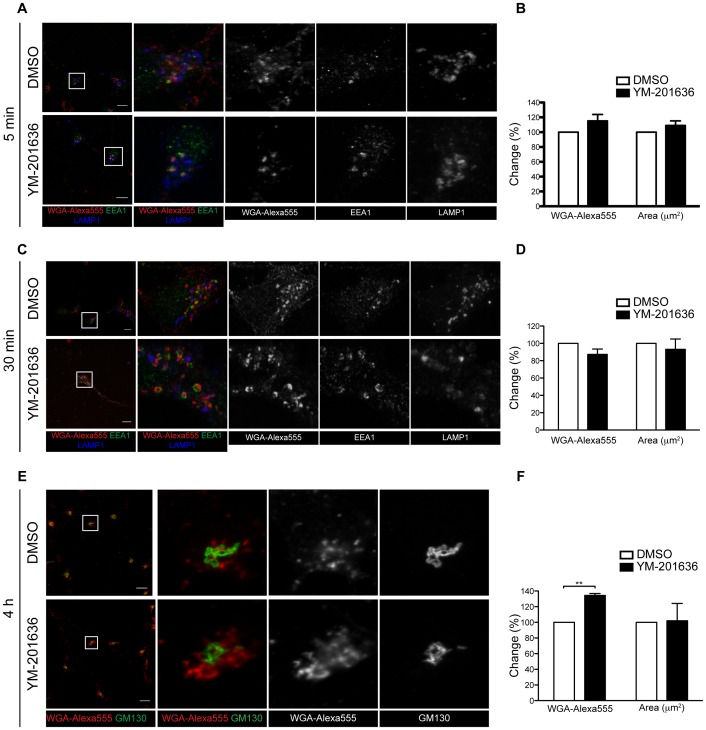
Effect of YM-201636 on WGA trafficking by immunocytochemistry. Primary hippocampal neurons were treated with DMSO or 1 µM YM-201636 for 4 h, then supplemented with 5 µg/ml WGA-Alexa555 for the final 5 min (A,B) or 30 min (C,D). Cells were fixed, immunolabelled for EEA1 and LAMP1, and imaged by confocal microscopy. (B,D) The intensity of WGA-Alexa555 per µm^2^ in the cell body and the total area of the cell body were determined in the YM-201636–treated cells relative to DMSO. (E,F) Primary hippocampal neurons were treated with DMSO or 1 µM YM-201636 in the presence of 5 µg/ml WGA-Alexa555 for the full 4 h. Cells were fixed, immunolabelled for GM130 and imaged by confocal microscopy. (F) The amount of WGA-Alexa555 fluorescent intensity in the cell body/µm^2^ of the YM-2016363 treated cells was determined relative to DMSO. The area of the cell bodies analysed was also determined (mean ± SEM, n = 3 independent experiments, 9–28 cells per experiment). Significances relative to DMSO **p<0.01. Scale bar = 10 µm.

As we had shown that inhibition of PIKfyve affects the size and morphology of endosomal/pre-lysosomal compartments, we next examined the effect of YM-201636 on endocytic trafficking in neurons. Using markers of three distinct endocytic trafficking pathways; retrograde transport to the Golgi complex (cholera toxin Β-subunit (CTB) [35]), endosomal recycling (transferrin (Tf) [36]), or transport to the late endosomal system (wheatgerm agglutinin (WGA)), we found no difference in the initial endocytosis, although there were some mild changes in the subsequent trafficking of these proteins by immunofluorescence microscopy (Figure 3, 4).

**Figure 5:**
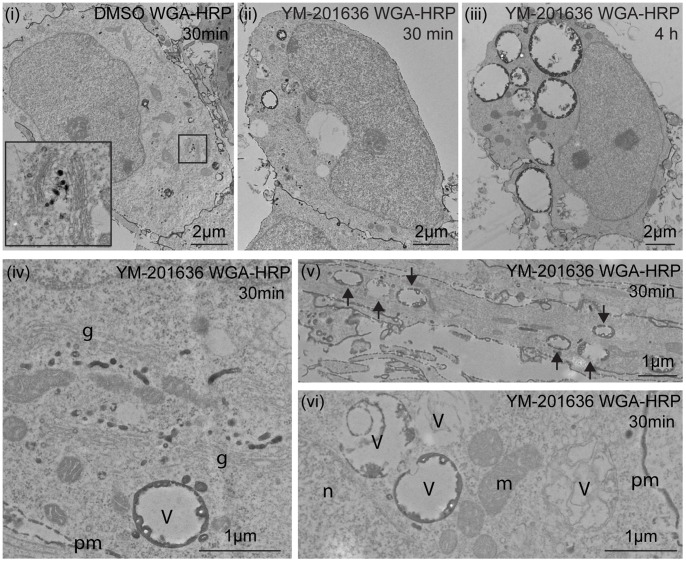
Ultrastructural analysis of the effect of YM-201636 on WGA trafficking. Primary hippocampal neurons were treated with (i) DMSO for 3.5 h and 10 µg/ml WGA-HRP for a further 30 min, (ii, iv–vi) 1 µM YM-201636 for 3.5 h and 10 µg/ml WGA-HRP for a further 30 min or (iii) 1 µM YM-201636 and 10 µg/ml WGA-HRP for 4 h. Cells were fixed, processed for DAB cytochemistry and imaged by electron microscopy. (i) In DMSO-treated cells WGA was identified in small vacuoles and vesicles. (ii) When WGA was added following inhibition of PIKfyve for 3.5 h, it was observed in a subset (∼45%) of large vacuoles. (iii) When WGA was continually present during the inhibition of PIKfyve, most (∼90%) of vacuoles contained DAB reaction product. (iv) Transport of WGA-HRP to the peri-Golgi region was unperturbed by treatment with YM-201636. (v) Vacuoles containing endocytosed WGA-HRP were also detected in neurites (arrows). (vi) In most cases vacuoles containing WGA-HRP appeared devoid of internal structures. g = Golgi complex, m = mitochondria, n-nucleus, V = vacuoles, pm = plasma membrane.

CTB was endocytosed into the cell normally (Figure 3A,C), however, a small but significant decrease in the amount that colocalised with GM130 in the Golgi region was detected (Figure 3A,B) suggesting partial inhibition in the latter steps of retrograde trafficking. For both Tf and WGA, there was a clear increase in the size of the endosomal compartments that these markers entered in YM-201636-treated cells, consistent with the effect of PIKfyve inhibition on the morphology of the early and late endosomes shown above. Transferrin normally recycles through early endosomes back to the cell surface [36]. To determine whether PIKfyve inhibition alters this trafficking pathway, the level of colocalisation between Tf and LAMP1 was determined (Figure 3D,E). Consistent with the decreased correlation between EEA1 and LAMP1 (Figure 2), there was also a reduction in colocalisation between Tf and LAMP1 (from 0.24±0.02 to 0.09±0.03, n = 26–37 cells from 2 independent experiments) following YM-201636 treatment. This data suggests that Tf is not mis-targeted to LAMP1 compartments following inhibition of PIKfyve, and is again consistent with the redistribution of LAMP1 compartments to more peripheral locations. Endocytosis of WGA also occurred normally with no change in the total intensity in the cell body following 5 and 30 min internalisation (Figure 4A–D). However, following a 4 h incubation period, a small but significant increase in the intensity of WGA was observed upon treatment with YM-201636 (Figure 4E,F). Inhibition of PIKfyve has been reported to prevent maturation of lysosomes [32], which may inhibit the degradation of WGA resulting in increased accumulation in late endosomal/lysosomal compartments. Analysis of WGA trafficking by electron microscopy revealed that in control cells endocytosed WGA-HRP accumulated in small vesicular, tubulovesicular and vacuolar (<0.5 µm) compartments, and was detected close to the Golgi complex, consistent with its transport through the endolysosomal system and retrograde traffic (Figure 5i). YM-201636 treatment for 4 h in the continual presence of WGA-HRP revealed that in addition to vesicular structures and retrograde traffic, over 80% of the enlarged vacuoles generated (>0.5 µm) also contained endocytosed WGA, suggesting that most vacuoles were derived, at least in part, from endocytic traffic (Figure 5iii). Furthermore, when neurons were supplemented with WGA-HRP for the final 30 min of a 4 h YM-201636 treatment, a significant proportion (∼25–50%) of the vacuoles again contained endocytosed WGA, demonstrating that traffic from the cell surface to a subset of vacuoles was maintained despite the altered morphology (Figure 5ii,iv–vi). Together these data suggest that the dominant site of action of PIKfyve in primary cultured neurons lies at the interface between prelysosomal and lysosomal compartments.

### PIKfyve Inhibition Dysregulates Autophagy in Neurons and Neuroendocrine Cells

**Figure 6:**
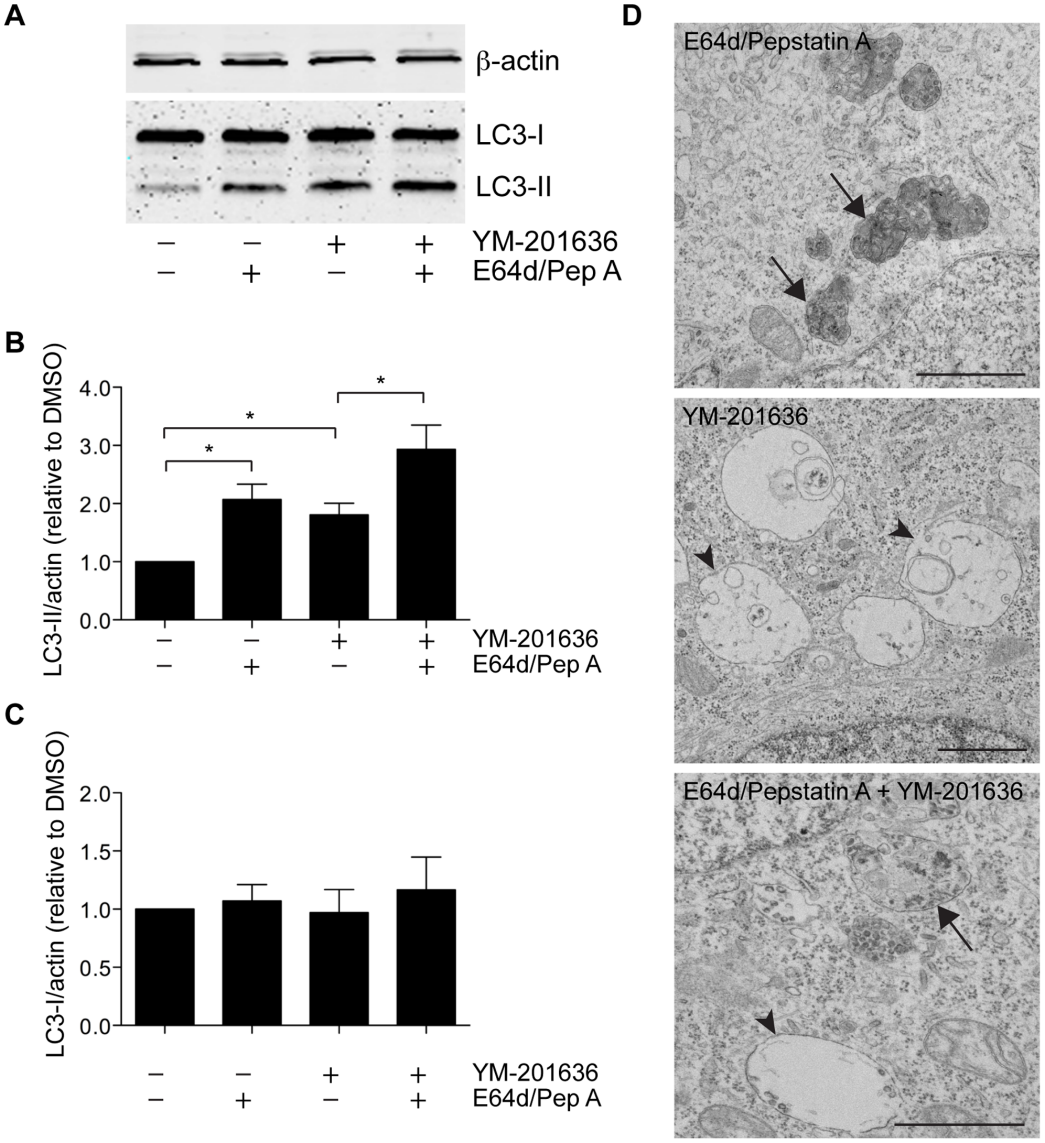
PIKfyve and lysosomal proteases inhibition augments LC3-II levels in cultured hippocampal neurons. (A) Primary hippocampal neurons were treated with DMSO or 1 µM YM-201636 for 4 h in the presence or absence of 10 µg/ml E64d and 10 µg/ml Pepstatin A. Samples were prepared for SDS-PAGE and immunoblotted for LC3 and ß-actin. The levels of LC3-II (B) and LC3-I (C) were normalised to ß-actin and quantified relative to DMSO alone. n = 4, mean ± SEM, *p<0.05 paired 1-tailed t-test. (C) Primary hippocampal neurons were treated with 10 µg/ml E64d/10 µg/ml Pepstatin A, 1 µM YM-201636 or 10 µg/ml E64d/10 µg/ml Pepstatin A +1 µM YM-201636 for 4 h, fixed and processed for electron microscopy. In all cases enlarged endolysosomal compartments were observed, however while inhibition of lysosomal proteases resulted in the formation of electron dense compartments with amorphous and membranous inclusions (arrows), inhibition of PIKfyve resulted in predominantly electronlucent compartments (arrowheads). Size bars = 1 µm.

While there was no change in the number of readily identifiable autophagosomal compartments at steady state upon PIKfyve inhibition, the morphology of a subset of the vacuolar compartments clearly suggested an autophagic component (Figure 2E). We therefore examined the effect of PIKfyve inhibition on microtubule-associated protein light chain 3 (LC3), an autophagosomal marker protein. The soluble form of LC3, LC3-I, undergoes post-translational modification by an ubiquitination-like reaction, in which an exposed C-terminal glycine is modified by phosphatidylethanolamine to generate the membrane-bound form, LC3-II, the levels of which can be used to measure autophagy [37]. To examine autophagy following PIKfyve inhibition, we measured the amount of LC3-II after 4 h and 22 h treatment with YM-201636. YM-201636-treated neurons exhibited a significant increase in the level of LC3-II (Figure 6A,B), while no change in the levels of LC3-I could be detected (Figure 6C). The increase in LC3-II was detected within 4 h, correlating temporally with the onset of vacuolation, and persisted through to 22 h, preceding the onset of cell death (Figure S3). Since intralumenal LC3-II is degraded by lysosomal proteases following fusion of the autophagosome with the lysosome [37], determining the level of LC3-II in the presence and absence of lysosomal protease activity can provide information regarding the delivery of autophagosomal content to the lysosome. As expected, inhibition of lysosomal proteases by E64d and pepstatin A in control neurons resulted in a significant increase in LC3-II levels (Figure 6A,B). Importantly, concomitant inhibition of PIKfyve and lysosomal proteases resulted in a further potentiation of LC3-II levels compared to treatment with either YM-201636 or E64d/pepstatin A alone. Importantly, inhibition of lysosomal proteases resulted in the formation of highly enlarged lysosomal compartments morphologically distinct from the vacuoles formed following PIKfyve inhibition, while simultaneous inhibition of PIKfyve and lysosomal proteases resulted in the concomitant formation of both types of compartments (Figure 6D), strongly suggesting that the primary affect of PIKfyve inhibition is not on lysosome proteolytic function.

**Figure 7:**
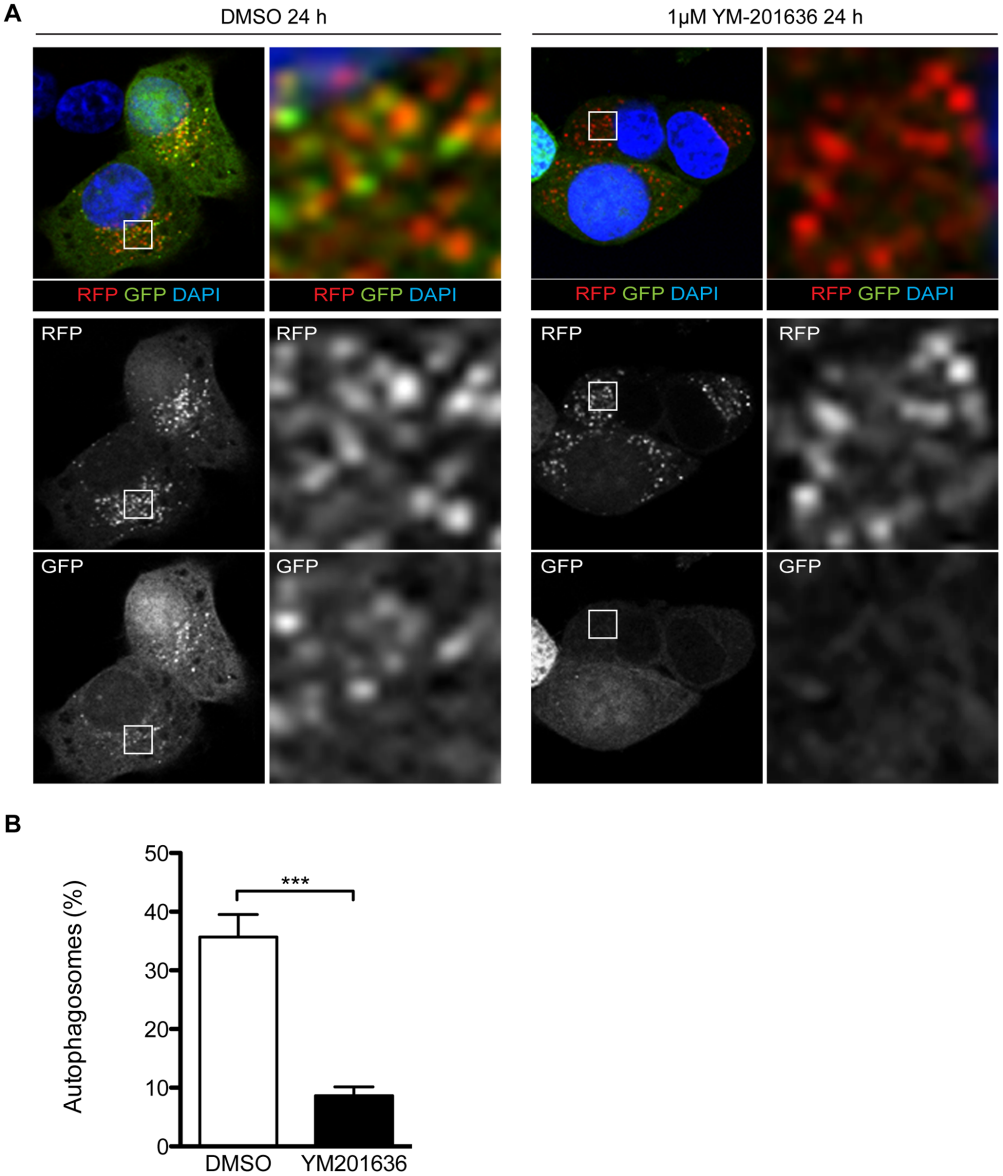
Processing of tf-LC3 in PC12 cells. (A) PC12/tfLC3 cells were treated for 24 h with 1 µM YM-201636 or DMSO, fixed and nuclei labelled using DAPI. The distribution and fluorescence of GFP and RFP were analysed by confocal microscopy. (B) The number of autophagosomes (determined by colabeling for GFP and RFP) was compared to the total number of RFP puncta, mean ± SEM, ***p<0.001 (n = 16–18 images from 2 independent experiments).

Since autophagy is a multistep process requiring sequential membrane trafficking and fusion reactions, the increased LC3-II levels observed in neurons could stem from a requirement for PIKfyve activity in one or more distinct regulatory steps including i) *de novo* formation of autophagosomes, ii) the rate of consumption of existing autophagosomes by the lysosomal system and/or iii) processing of intralumenal LC3-II in the autolysosome. To begin to investigate these individual steps, we used neurosecretory PC12 cells engineered to stably express the reporter construct GFP-RFP tandem-fluorescent LC3 (tfLC3). tfLC3 can be used to distinguish between autophagosomes (which retain GFP fluorescence) and acidified autolysosomes (in which the external tfLC3 has dissociated and the GFP tag of luminal tfLC3 is quenched by the acidic pH) [22]. Inhibition of PIKfyve by YM-201636 in PC12 cells phenotypically mimicked the effect observed in hippocampal neurons, including increased vacuolation as observed by electron microscopy, and an increase in LC3-II (data not shown), confirming that they were an appropriate model. Notably, however, PC12 cell survival was not affected by YM-201636 over a 24 h period, a difference that enabled us to further probe potential autophagic trafficking defects. PC12/tfLC3 cells were found to contain a large number of autophagosomes (Figure 6A), identified by the presence of coincident GFP/RFP fluorescence, in addition to autolysosomes, which displayed RFP fluorescence only (Figure 7A). Following treatment with YM-201636 we found that while the total number of RFP-positive autophagic compartments was unaltered (43.02±6.40/cell in DMSO-treated vs. 40.17±5.03/cell in YM-201636-treated, n = 16–18 cells) there was a highly significant reduction in the proportion of these compartments that were also GFP positive (Figure 7B). This could potentially be due to either a decrease in the *de novo* formation of autophagosomes, an increase in the rate of consumption of autophagosomes by the lysosomal system, or increased acidification of prelysosomal, autophagic compartments. Together, these data suggest that PIKfyve activity is an important regulator of autophagy, however, they further suggest that PIKfyve activity could be required at several distinct steps of the autophagic process.

## Discussion

In this study, we have used the PIKfyve inhibitor YM-201636 to investigate the effects of acutely reducing PtdIns(3,5)*P*_2_ levels in hippocampal neurons in culture. Consistent with studies in non-neuronal cell lines [3], [6], [16], [17], directly inhibiting PIKfyve activity results in the swelling of endocytic compartments and disruption of endomembrane transport, which in neurons precedes significant levels of apoptosis-independent cell death. PIKfyve is known to play a key role in the regulation of a number of cellular processes, including trafficking through the endolysosomal system, exocytosis, ion channel regulation and autophagy [3]–[8], [10]. Despite morphological alterations in the endolysosomal system, we found that the endocytosis of marker proteins was largely unaffected. In contrast, we observed significant dysregulation of the autophagosomal and lysosomal systems, suggesting that alterations in the autophagic pathway in neurons could underlie the survival defect that results from PIKfyve inhibition.

### Endocytosis, Trafficking and Signalling

Vacuolation of cells upon disruption of PIKfyve activity, whether by inhibitors, siRNA-mediated knock down or over-expression of a dominant-negative kinase-dead PIKfyve, has been reported in a variety of cell lines [3], [16], [17], [34]. Consistent with these results, one of the earliest phenotypes we detected upon acute inhibition of PIKfyve in cultured hippocampal neurons was the formation of numerous enlarged vacuoles. These vacuoles labelled for markers of early endosomes (EEA1) and lysosomes (LAMP1), indicating an endosomal origin, which is also consistent with studies in non-neuronal cell lines [3], [16], [17], [34]. Despite the clear enlargement of endosomal compartments, their specific characteristics (canonical resident protein distribution and trafficking itineraries of receptors) appeared normal. However, there were some notable differences in the endocytic trafficking, but not endocytosis itself, of marker proteins including CTB and WGA. These data are in good agreement with previous studies [3], [16], [17]. Consistent with studies in non-neuronal cells (17), we observed a slight decrease in the amount of CTB undergoing retrograde trafficking to the Golgi complex. Furthermore, WGA showed increased intracellular accumulation after prolonged PIKfyve inhibition consistent with the finding that lysosomal maturation is blocked [32]. Whether PIKfyve inhibition specifically alters the trafficking, targeting or silencing of neuronal survival signals is currently unknown. However, previous studies of epidermal growth factor receptor trafficking have shown PIKfyve inhibition to have little [3], or no [16], [17], effect on this pathway. Furthermore, in *D. melanogaster* carrying mutations in the PIKfyve homologue Fab1, the signalling and silencing of endocytosed cell survival factors is unimpaired despite the altered endosomal morphology [38]. Although these data suggest that neuronal cell death is unlikely to result from alterations in the trafficking or signalling of growth factors, a more targeted approach, specifically looking at the trafficking of key survival factors will be needed to further assess this possibility.

### Neuronal Cell Death Mechanisms

The contribution of apoptotic cell death pathways to the development of neurodegenerative diseases has been well characterised [28], [29]. However, less well understood is the contribution of alternative, non-apoptotic mechanisms including autophagic cell death [39] and programmed necrosis (necroptosis) [40]. In the current study, we have shown that neuronal cell death associated with defective PIKfyve activity in cultured embryonic hippocampal neurons is independent of caspase activity. This suggests that the neurodegenerative phenotype observed in mice deficient in Fig4 or Vac14, and in Charcot-Marie-Tooth disease type 4J disease in humans, is unlikely to be mediated by apoptosis. Rather our data and that of others [20] point towards dysregulation of the autolysosomal system as a potential underlying cause of neuronal death *in vivo*. Previous studies have suggested direct links between the activity of PIKfyve and dysregulation of autophagy in both mammalian cells [3], [6] and model organisms such as *C. elegans* and *D. melanogaster* [32], [38], [41]. Consistent with dysregulated autophagy *in vivo*, there is an increase in LC3-II levels in the brains of Fig4-deficient and Vac14 mutant mice [20], although the cellular origin of this was not investigated. While our own studies point to a clear dysregulation of autophagy in both neurons and neuroendocrine cells upon inhibition of PIKfyve, we have additionally identified defects in the late lysosomal system, suggesting that PIKfyve could act at multiple points within the autolysosomal system. A more detailed dissection of the individual trafficking steps affected by inhibition of PIKfyve is warranted, as is an analysis of any secondary effects leading from loss of PtdIns(3,5)*P*_2_.

Finally, it is also possible that *in vivo*, other mechanisms could be contributing to the observed neuronal cell death in mice with reduced levels of PtdIns(3,5)*P*_2_. For example, PIKfyve has been implicated in the regulation of other neuronal process including neurosecretion [8], protection from glutamate-induced excitotoxic cell death by regulation of CaV1.2 degradation at the lysosome [10], and the control of post-synaptic function through recycling of AMPA receptors [42]. Furthermore, recent studies using targeted re-expression of Fig4 into either neurons or glia of Fig4^−/−^ mice have shown that while loss of PIKfyve activity in neurons is causative for spongiform degeneration, dysregulation of autophagy is most associated with glial cells [15]. In the present study, we have shown that inhibition of PIKfyve using the inhibitor YM-201636 dysregulates autophagy and promotes neuronal cell death in primary hippocampal neurons in culture. Future investigations into the PtdIns(3,5)*P*_2_ effectors [43] promoting neuronal cell death are warranted. The effect of PIKfyve inhibition in established neuronal cultures remains to be determined. The possibility of off-target effects of YM-201636 cannot be ruled out and further studies using viral delivery methods to knockdown PIKfyve expression will establish this.

In conclusion, our data point to a fundamental requirement for PtdIns(3,5)*P*_2_ in the survival and development of hippocampal neurons, and further suggest that alterations in the flux of material through the autophagosomal/lysosomal system could contribute to the mechanism of neuronal cell death observed in response to PIKfyve inhibition. Further studies designed to delineate the precise function(s) of PIKfyve in integrating regulation of the endosomal and autophagosomal systems in neurons will provide essential new insights into this important nexus in neuronal survival.

## Supporting Information

Figure S1 **Vacuolation and cell death in glial cells.** (A) Still images of glial cells within the neuron preparation treated with 1 µM YM-201636 for the times shown and imaged by phase contrast. (B) Percentage of dead glial cells, n = 4. (C) Percentage of vacuolated glial cells, n = 4. All results show mean ± SEM. Circle = DMSO, square = YM-201636. Significances relative to DMSO *p<0.05, **p<0.01, ***p<0.001.
(TIF)

Figure S2 **Morphological classification of vacuoles and autophagic/lysosomal compartments.** Primary hippocampal neurons were treated with 1 µM YM-201636 for 4 h, fixed and processed for electron microscopy. Autophagosomes presenting a double membrane and luminal content indistinguishable from cytosol were classified as immature (AVi), whereas autophagic compartments presenting a double membrane with heterogeneous, electron-dense luminal content were classified as degradative (AVd). Lysosomes were classified by a single limiting membrane and electron-dense lumen (shown), which could also include membrane sheets and lamellae (not shown).
(TIF)

Figure S3 **Increased LC3 levels in YM-201636-treated neurons.** (A) Immunoblotting for LC3 and ß-actin in primary hippocampal neurons treated with DMSO or 1 µM YM-201636 for 4 h or 22 h, or maintained in serum-free medium (Starved) for 4 h. (B) The level of LC3-II was normalised to total LC3 and quantified for each time point relative to the control treatment, n = 4, mean ± SEM, *p<0.05.
(TIF)

Movie S1 **Time-lapse imaging of primary hippocampal neurons – control.** Cultured hippocampal neurons were grown for 2 days in a 24-well plate before imaging with an inverted Axio Observer microscope and AxioVision software (Zeiss). The neurons were contained in a 37°C, 5% CO_2_ chamber during time-lapse imaging. DMSO was added at 0 min (immediately following frame 1) and phase contrast images were subsequently taken every 10 min over 48 h.
(MOV)

Movie S2 **Time-lapse imaging of primary hippocampal neurons –1** **µM YM-201636.** Cultured hippocampal neurons were grown for 2 days in a 24-well plate before imaging with an inverted Axio Observer microscope and AxioVision software (Zeiss). The neurons were contained in a 37°C, 5% CO_2_ chamber during time-lapse imaging. YM-201636 (1 µM) was added at 0 min (immediately following frame 1) and phase contrast images were subsequently taken every 10 min over 48 h.
(MOV)
