# Supplementary figures and images for: Inhibition of PIKfyve by YM-201636 Dysregulates Autophagy and Leads to Apoptosis-Independent Neuronal Cell Death
Source: PLoS One. 2013 Mar 27;8(3):e60152. doi: 10.1371/journal.pone.0060152 (PMC3609765; doi:10.1371/journal.pone.0060152)

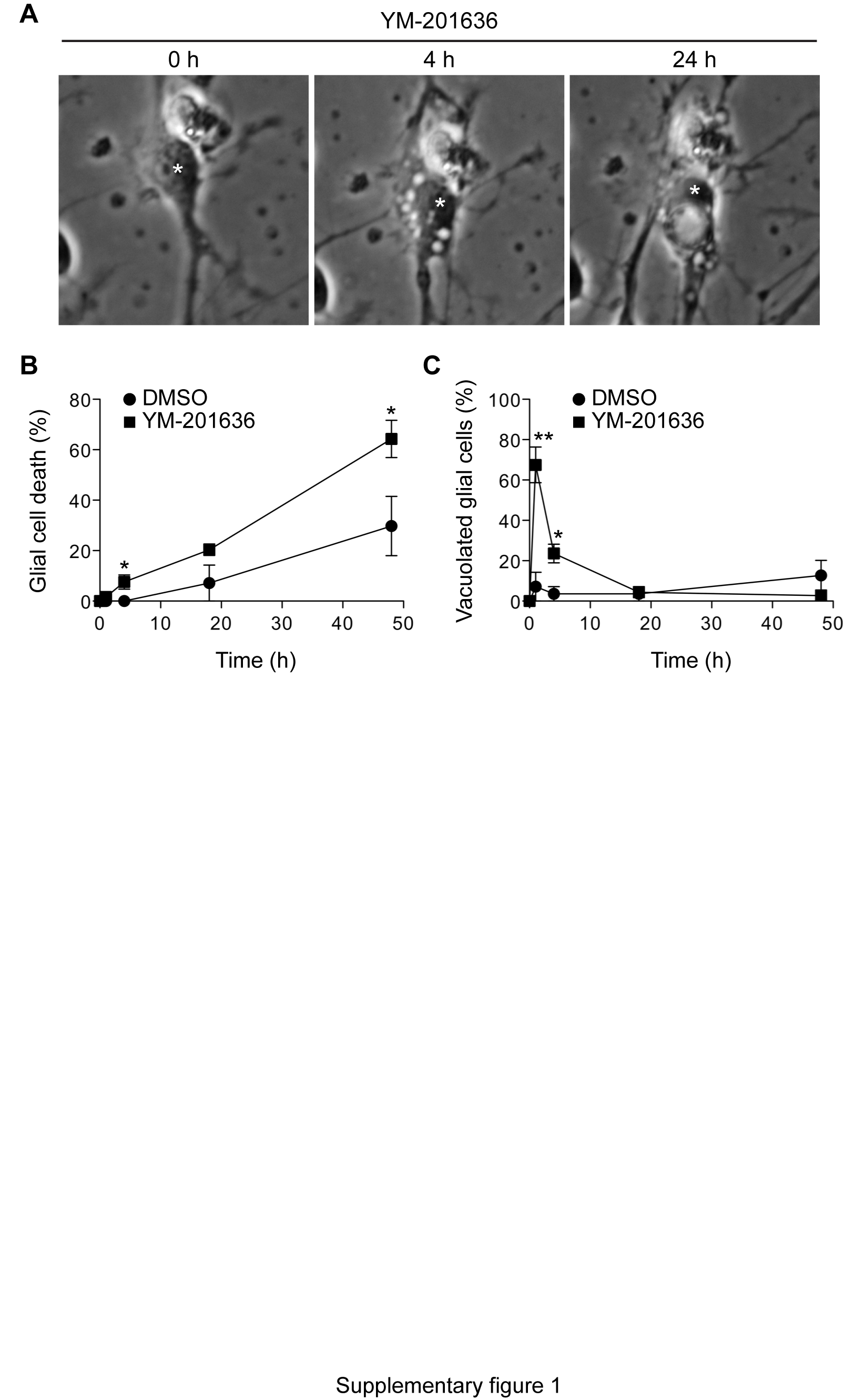

Supplement: Figure S1 — Vacuolation and cell death in glial cells. (A) Still images of glial cells within the neuron preparation treated with 1 µM YM-201636 for the times shown and imaged by phase contrast. (B) Percentage of dead glial cells, n = 4. (C) Percentage of vacuolated glial cells, n = 4. All results show mean ± SEM. Circle = DMSO, square = YM-201636. Significances relative to DMSO *p<0.05, **p<0.01, ***p<0.001. (TIF) [file pone.0060152.s001.tif]

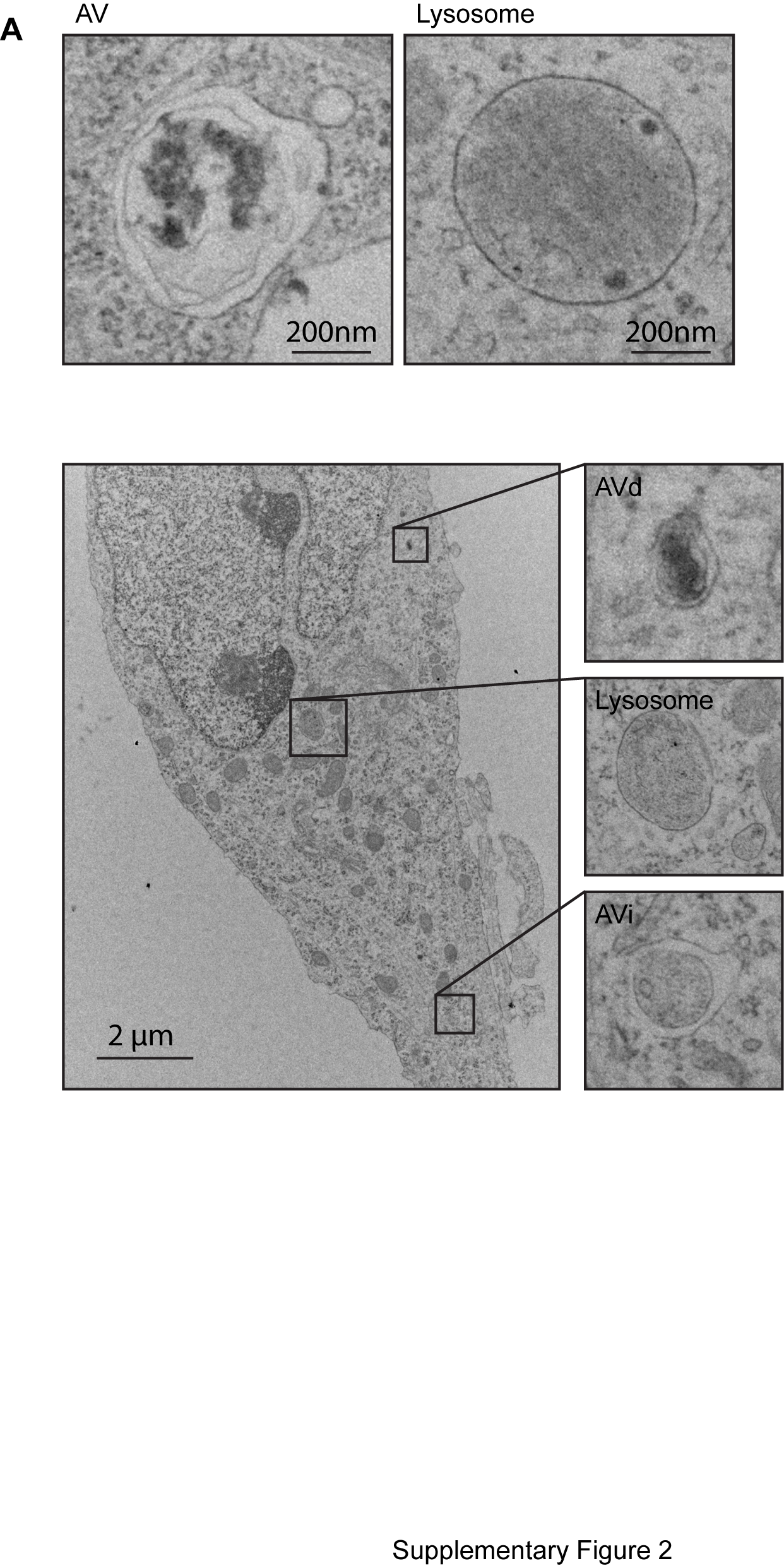

Supplement: Figure S2 — Morphological classification of vacuoles and autophagic/lysosomal compartments. Primary hippocampal neurons were treated with 1 µM YM-201636 for 4 h, fixed and processed for electron microscopy. Autophagosomes presenting a double membrane and luminal content indistinguishable from cytosol were classified as immature (AVi), whereas autophagic compartments presenting a double membrane with heterogeneous, electron-dense luminal content were classified as degradative (AVd). Lysosomes were classified by a single limiting membrane and electron-dense lumen (shown), which could also include membrane sheets and lamellae (not shown). (TIF) [file pone.0060152.s002.tif]

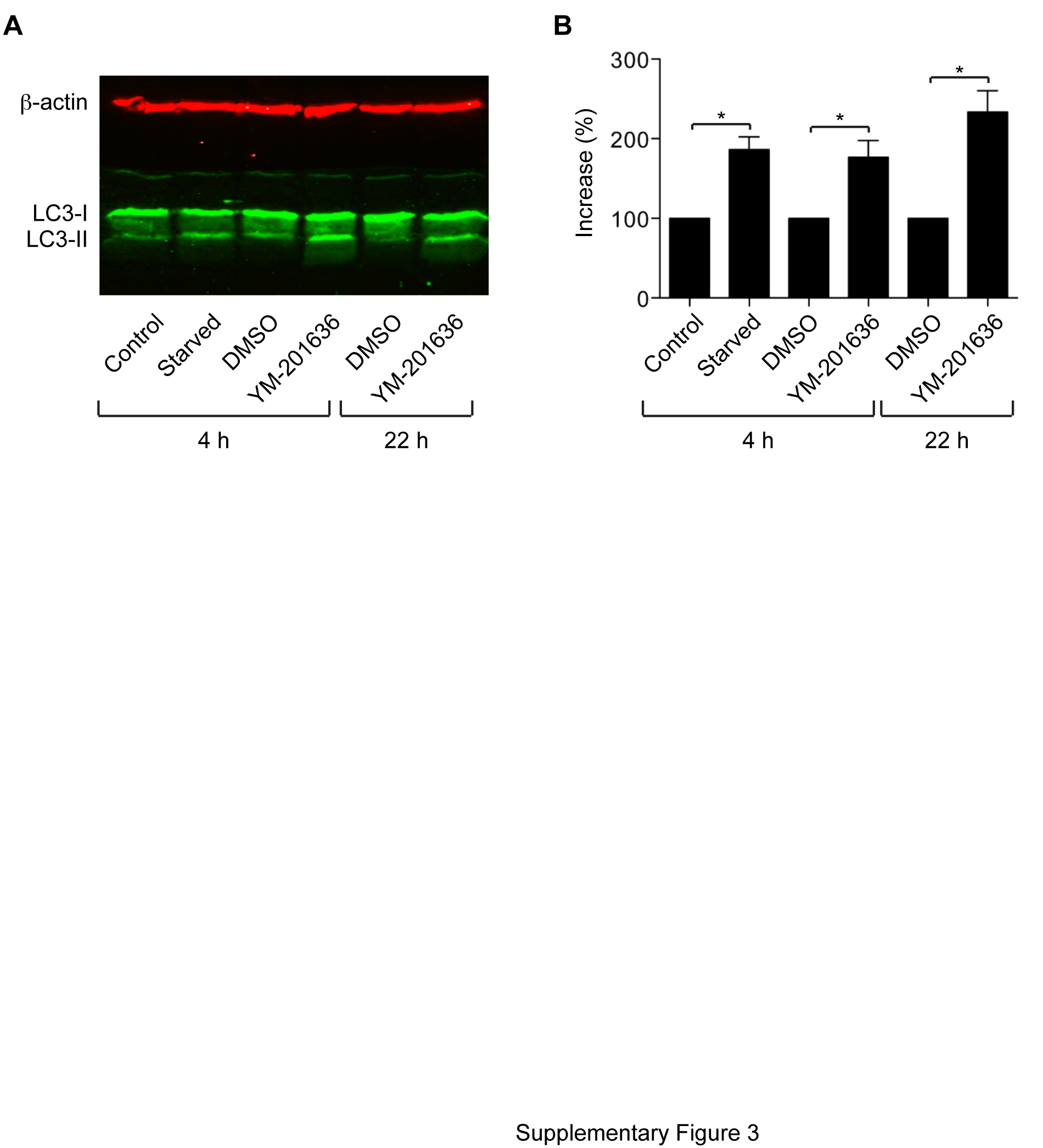

Supplement: Figure S3 — Increased LC3 levels in YM-201636-treated neurons. (A) Immunoblotting for LC3 and ß-actin in primary hippocampal neurons treated with DMSO or 1 µM YM-201636 for 4 h or 22 h, or maintained in serum-free medium (Starved) for 4 h. (B) The level of LC3-II was normalised to total LC3 and quantified for each time point relative to the control treatment, n = 4, mean ± SEM, *p<0.05. (TIF) [file pone.0060152.s003.tif]
